# Food Safety and Health Concerns of Synthetic Food Colors: An Update

**DOI:** 10.3390/toxics12070466

**Published:** 2024-06-27

**Authors:** Petra Amchova, Filip Siska, Jana Ruda-Kucerova

**Affiliations:** 1 Department of Pharmacology, Faculty of Medicine, Masaryk University, Kamenice 5, 625 00 Brno, Czech Republic; pamchova@med.muni.cz (P.A.); siska@med.muni.cz (F.S.); 2 Oncology Department, Hospital of Ceske Budejovice, B. Nemcove 585/54, 370 01 Ceske Budejovice, Czech Republic

**Keywords:** toxicity, food dye, color, safety, ADI

## Abstract

The toxicity of food additives is widely studied and concerns many consumers worldwide. Synthetic food colors are often considered an unnecessary risk to consumer health. Since the European Food Safety Authority’s (EFSA) re-evaluation between 2009 and 2014, the body of scientific literature on food colors has grown, and new evaluations are being published by the Joint FAO/WHO Expert Committee on Food Additives (JECFA). Therefore, this narrative review aims to review the toxicological data that have become available since 2014. The reviewed colors are Quinoline Yellow, Sunset Yellow, Azorubine, Amaranth, Ponceau 4R, Erythrosine, Allura Red, Patent Blue, Indigo Carmine, Brilliant Blue FCF, Green S, Brilliant Black, Brown HT, and Lithol Rubine BK. Tartrazine was not included in this paper; the overwhelming amount of recent data on Tartrazine toxicity requires more space than this review can provide. The issues regarding the toxicity of synthetic food colors and real population exposures are being regularly examined and reviewed by relevant authorities, such as the EFSA and JECFA. The current ADI limits set by the authorities are mostly in agreement, and they seem safe. However, the EFSA and JECFA assessments of some of the colors are more than a decade old, and new evidence will soon be required.

## 1. Introduction

Food colors are substances added to food mainly to introduce color, prevent a loss of color due to environmental conditions, or enhance naturally occurring colors. In recent decades, increasing attention has been paid to the potential toxicity of soluble synthetic colors, particularly azo dyes [1,2]. All food additives, including colors, that are used in the European Union must be reviewed by the European Food Safety Authority (EFSA), which belongs to the European Commission. Between 2009 and 2016, an EFSA panel re-evaluated the safety of all forty-one previously authorized food colors. We summarized the available evidence and changes in the acceptable daily intake (ADI) values of azo dyes in 2015 [1] while the EFSA was finishing its re-evaluation. However, concerns regarding the potential toxicity of synthetic colors remain, even as new studies are being published. The current reports are still not exhaustive, but new and important findings are available. Therefore, the aim of this review is to provide an update on the current knowledge of toxicity issues related to azo dyes. Tartrazine was not included in this review because the abundance of data available for this chemical makes it warrant its own separate review. For easier orientation of the text, we include a glossary of the most commonly used terms in the field of food safety regulations (Table 1).

For this review, we searched the Google Scholar database for the names of the colors together with “toxicity”, with the timeframe limited to papers published in 2014 or later. Then, we manually reviewed the results and used those studies that were relevant for this review. We only included papers written in English that focused on toxicity issues. We did not use any strict inclusion/exclusion criteria to assess the quality standards of the reports. Our aim was to gather all relevant evidence available to date. Only findings supported by statistically significant differences in the data were included. Despite the usual way of summarizing evidence by presenting the results in tables, in this case, there were not many comparable studies for each color. Therefore, we opted for descriptions in the text.

## 2. Toxicity Evaluation of Azo Dyes

The following is an overview of the toxicity of individual synthetic colors approved in the EU. An overview of their chemical structures, current ADIs, and years of the last EFSA and Joint FAO/WHO Expert Committee on Food Additives (JECFA) re-evaluations are summarized in Table 2. 

### 2.1. E 104 FLAVUM QUINOLINI, Quinoline Yellow

Quinoline Yellow is one of the colors raising the greatest health concerns, including about the potential effects on children’s behavior. This is reflected in the last EFSA re-evaluation, in which the ADI was lowered from 10 mg/kg to the current 0.5 mg/kg. The panel warned that the actual consumption of the color additive usually exceeds the new ADI value, with a theoretical maximum daily exposure of 8.1 mg/kg/day for adults and 13.1 mg/kg/day for 3-year-old children [3]; however, these results were not confirmed in a later study [26]. Similarly, in 2011, the JECFA established a temporary ADI of 5 mg/kg, withdrawing the previous value of 10 mg/kg [6], and set the new ADI of 3 mg/kg in 2016 [4]. The European Commission requested the EFSA perform a refined food exposure assessment for Quinoline Yellow. To this end, the EFSA collected usage levels for 6 out of 28 food categories in which the color is authorized and concluded that the highest dietary intake is in toddlers and children—up to 0.40 mg/kg of body weight per day [26]. Hence, we can summarize that the actual consumption seems to be under the current ADI set by the EFSA.

There are some new studies relevant to the safety of Quinoline Yellow. An in vitro study assessed the propensity of the color to induce conformational changes in proteins; hen egg-white lysozyme was selected as a model protein [27]. Quinoline Yellow induced the formation of lysozyme aggregates at physiological pH 7.4 at a concentration of 25–100 mM [27]. The same team proved the ability of Quinoline Yellow to bind to myoglobin at pH 3.5, resulting in protein aggregation, but this interaction did not occur at pH 7.4 [28]. These in vitro results represent merely a proof of concept; however, such protein aggregates can be toxic, and this issue deserves future attention.

Furthermore, in vitro, Quinoline Yellow was shown to be a potent agonist of the aryl hydrocarbon receptor, a *CYP1A1* gene inducer, and an inhibitor of estrogen receptor signaling in an aryl-hydrocarbon-receptor-dependent manner [29]. These findings were inconclusive but suggest the color may have a potential for endocrine disruption. 

After Quinoline Yellow showed mutagenic potential in several in vitro studies [1], the EFSA and JECFA reviewed a number of genotoxicity studies and concluded that Quinoline Yellow does not raise genotoxicity concerns. A recent in vitro study showed that the dye could modulate 21 genes related to the DNA repair processes [30], which may raise a concern. However, this study was not a test-guideline genotoxicity study, as recommended by food regulators. Lastly, an in vitro study assessing the interaction of Quinoline Yellow with P-glycoprotein did not indicate any such interaction [31].

Taken together, ongoing research on the potential health risks related to Quinoline Yellow indicates that this topic is far from concluded, and future studies will be necessary to reach a final consensus regarding its use as a food additive.

### 2.2. E 110 FLAVUM ORANGEATUM, Sunset Yellow

Multiple authorities have evaluated the toxicity of Sunset Yellow because of concerns about its wide use in the culinary and pharmaceutical industries. The additive can cause the release of histamine, potentially triggering various allergies and intensifying symptoms of asthma [32,33,34]. In 2009, the EFSA established a temporary ADI of 1 mg/kg of body weight per day; however, this temporary ADI was revised in 2014, establishing a new ADI of 4 mg/kg per day [5]. The JECFA had reached a similar conclusion before the EFSA and set the same ADI level in 2011 [6]. 

In the last decade, several new studies have been conducted to examine the potential toxicity of Sunset Yellow, mainly focusing on its potential teratogenic properties. Models using zebrafish (*Danio rerio*) embryos exposed to graded concentrations showed concentration-dependent toxicity. The concentration of 1 mM induced morphological deformities such as microphthalmia, pericardial edema, yolk sac edema, and spinal curvature. Higher concentrations (10–50 mM) led to higher mortality due to increased cellular apoptosis in the cardiac region [35,36]. Another study used three doses (200, 1000, and 2000 nanograms) of Sunset Yellow to examine its possible teratogenic effects on chicken embryos. Toxicity was evident with all three doses, resulting in necrosis of hepatocytes and multiple degenerative changes in the kidneys [37]. Both models met OECD guideline standards for teratogenicity testing. A similar design was used to investigate the effect of Sunset Yellow on the development of immune system organs. Exposed embryos showed a greater increase in spleen volume, increased eosinophil ratios, developmental retardation in the thymus, and less lymphocyte accumulation in the bursa of Fabricius, suggesting disruption of immune system development [38]. The toxicity of high doses of Sunset Yellow was examined in germ cells of male rats exposed to 500, 1000, and 1500 mg/kg body weight/day of the dye for 28 days, and it was found that the sperm quality was not affected [39]. Another study used direct incubation of human germ cells with Sunset Yellow. While low concentrations (50 and 100 μg/mL) did not affect the tail DNA % and tail length of sperm, the 200 μg/mL concentration showed a significant increase in tailed DNA %, suggesting a potential mutagenic effect [40]. 

Direct genotoxic effects were observed in meristematic cells of *Brassica campestris* L. exposed to different concentrations of Sunset Yellow (1%, 3%, and 5%). All treated cells showed decreased mitotic indexes and higher DNA abnormalities compared to the control group, and these effects increased with concentration [41]. These results are in accordance with a study focusing on the exposure of human lymphocytes to Sunset Yellow. Concentrations of 4 and 8 mM significantly increased the frequencies of cells with chromosome aberrations [42]. Genotoxicity was also assessed in female rats exposed to Sunset Yellow and sodium benzoate for 12 weeks. The results were similar to previous experiments; however, the lowest concentration used was 5 mg/kg of body weight, and the highest was 200 mg/kg, exceeding the current ADI [43]. 

Furthermore, the potential hepatotoxicity of Sunset Yellow was examined. Rats exposed to the mixture of Sunset Yellow, Metanil Yellow, and Tartrazine for 30 days showed a significant increase in the concentration of serum proteins, serum albumin, serum alkaline phosphates, and hepatic malondialdehyde and decreased activity of superoxide dismutase, glutathione, and catalase in liver tissue compared to the control group. However, concentrations of the mixture were 25–75 mg/kg of body weight/day, many times higher than the ADI [44]. Khayyat et al. presented similar findings when rats were exposed to Sunset Yellow at a dose of 2.5 mg/kg body weight/day for 30 days. Moreover, the data showed a decrease in Bcl2 expression and an increase in COX2 expression, as well as histopathological aberrations in the liver and kidney, suggesting oxidative-stress-mediated renal and hepatic toxicity [45]. The inflammatory response was also altered in intestinal epithelial organoids treated with 2 μg/mL of Sunset Yellow, inhibiting the proliferation and disturbing the differentiation of small intestinal epithelial cells. The exacerbation of intestinal inflammation by Sunset Yellow was confirmed in the dextran sulfate sodium-induced intestinal inflammation model in C57BL/6 mice treated with 40 mg/kg of body weight/day for 7 days [46]. 

Exposure to Sunset Yellow can also promote hematological changes. Rats exposed to a combination of Sunset Yellow and sodium benzoate over 12 weeks showed a decrease in hemoglobin, red blood cell count, and white blood cell count. This effect was concentration-dependent, and the minimum concentrations for Sunset Yellow and sodium benzoate were close to the ADI limits at 5 mg/kg of body weight and 10 mg/kg of body weight, respectively [47]. These findings were supported by Elbanna et al., who exposed rats to Sunset Yellow or its metabolites for 90 days. However, this study also showed that coadministration of azo dye with lactic acid bacteria decreased the inflammation and histopathological alterations in the kidney, liver, spleen, and small intestine [48].

Taken together, the current ADI seems to be a safe limit based on the available evidence, although higher doses can induce multiple toxic effects. 

### 2.3. E 122 AZORUBINUM, Azorubine

Several authorities have conducted multiple evaluations of Azorubine, and the original ADI has not changed since 1983. The EFSA’s Scientific Opinion from 2009 raised concerns about the intake in children aged 1 to 10 years possibly exceeding the ADI. However, in 2015, the refined exposure scenarios showed that the ADI was not exceeded by any of the population groups at intermediate or higher exposure levels [49].

Combining Azorubine and Sunset Yellow creates an orange-red dye that is applied in cosmetics and the food industry. The toxicity of this combination was assessed in a study using rats exposed to these two colors for 30 days. The findings demonstrated a detrimental effect on antioxidant status, inflammatory biomarkers (TNFα, IFNγ, IL1β, IL6, COX-2, iNOS, and NFκB/p65), biochemical enzymes, and liver histology. The toxicity was found to be dose-dependent, but very high doses were used—5, 25, 150, and 300 mg/kg [50]. Notably, administering Azorubine separately has been shown to have the same effect on hematological, immune, and histopathological changes in several organs in rats [48] and mice [51]. However, again, these results were obtained after chronic administration of very high doses, which is irrelevant for assessing the potential toxicity of food additives.

Azorubine has a certain potential to cause allergic reactions in predisposed individuals. For example, a hypersensitivity skin reaction and recurrent fixed drug eruption were reported in a case study of a child who had taken a medicine containing this azo dye [52]. A small prospective clinical study based on patch testing reported Azorubine as a contributing factor to the development of recurrent aphthous stomatitis [53]. However, this study has limited value—skin patch tests suggest sensitization to a substance but do not provide evidence for a food allergy response. Furthermore, Azorubine has a potential inhibitory effect in high and moderate concentrations on the intracellular production of reactive oxygen species in vitro. This effect may contribute to inflammatory processes or hypersensitivity of the organism [54]. Other studies have confirmed the pro-inflammatory effect of Azorubine due to augmentation of LTB4 production and increased synthesis of F2-isoprostanes in human neutrophils, which results in oxidative stress [55], an increase in malondialdehyde production, and reductions in glutathione, superoxide dismutase, and catalase expression in rats [56]. Azorubine, at both high concentrations and those close to the ADI, may cause increased cell apoptosis based on an observed increased expression of MAPK8 in mice [57].

Azorubine showed a negative effect on fertility in male rats at doses above the ADI. Specifically, it exerted degenerative changes and atrophy, gradually decreasing sperm count and reducing the expression of other fertility biomarkers [58]. 

An exploratory study showed dose-dependent degenerative changes in zebrafish larvae after Azorubine exposure. The no-observed-effect concentration (NOEC) was recorded to be 5 ppm, and the median lethal concentration (LC50) was 1230 ppm, when administered for 96 h per fertilization [59]. The genotoxicity of Azorubine was assessed using root meristematic cells of *Allium cepa*. Azorubine caused a significant reduction in the mitotic index and produced different kinds of chromosomal aberrations in these cells [60]. However, the relevance of these findings for human consumption is questionable.

Interestingly, Azorubine can bind to lysozymes and inhibit amyloid fibrillogenesis, exerting a defibrillating effect in the lysozymes. Therefore, it could play a role in the treatment of amyloid-related diseases [61].

Taken together, despite low intake as a food additive, Azorubine may represent a health issue. Therefore, its use shall be closely monitored, and the authorities will soon evaluate new evidence.

### 2.4. E 123 AMARANTHUM, Amaranth

Amaranth was repeatedly evaluated by the JECFA in 1972, 1975, 1978, and 1984 and by the EU Scientific Committee for Food (SCF) in 1976, 1979, and 1983. The JECFA and the SCF established different ADIs of 0.5 and 0.8 mg/kg, respectively. Amaranth’s use as a food additive was banned in the USA in 1976 because of concerns regarding its potential risks to human health. It is mainly used as a color for aperitif drinks and wines, which explains why exposure in toddlers and children is very low. At the same time, consumption in adults can exceed the ADI by several hundred percent in some scenarios [9]. Pharmacokinetic studies are not available in humans, but studies in rodents indicate little absorption of Amaranth (2.8% in rats) after oral administration [62]; therefore, the majority remains in the intestinal content. The azo-linkage of the dye can be broken during the passage, and metabolites are likely formed by intestinal bacteria. The proportions of the unchanged color and its metabolites in feces are variable. Amaranth is metabolized in the blood, where azo-linkage reduction and the formation of several metabolites (naphthionic acid being the main one) occur; these are then excreted in feces and urine [9]. There is no evidence of accumulation in any tissues in several species. However, though the data cover only 3 days of exposure [63], the color was found to penetrate several organs (lung, heart, and liver, but not the brain) and fetuses [64,65,66]. The toxicity of this color was assessed by several in vitro and in vivo studies, showing no evidence of genotoxicity [9,67] or carcinogenicity [9]. No reports of severe hypersensitivity are available. Developmental toxicity has been tested thoroughly in many studies for several species. As a result of developmental reports plus one 2-year-long exposure study, it was possible to define a NOAEL of 15 mg/kg of body weight per day for Amaranth. Therefore, by applying an uncertainty factor of 100, the EFSA defined a new ADI of 0.15 mg/kg [9]. A new study exploring the potential developmental toxicity of azo dyes using zebrafish embryos concluded that their positive findings occurred at concentrations higher than those expected in human food exposure [35].

Following the completion of the Scientific Opinion, the EFSA conducted a refined exposure assessment for Amaranth. This assessment did not confirm the previous concern about high exposure in adults consuming alcoholic aperitif drinks. Conversely, the dietary intake under any assessed scenario in any population did not exceed the current ADI [68]. 

Recently, a biophysical study evaluated a potential interaction of Amaranth with hemoglobin, showing the binding potential of the dye and following the conformational changes to the protein, suggesting a potential mechanism of toxic effect [69]. Based on this finding, it is evident that continued research, evaluation, and regulatory control of this dye is necessary to assess its potential risks for consumers. The EFSA published its Scientific Opinion on this color in 2009, while the JECFA published its version in 1983. Hence, new evaluations are needed. 

### 2.5. E 124 RUBOR PONCEAU, Ponceau 4R

In its last re-evaluation, the EFSA lowered the ADI for Ponceau 4R from 4 mg/kg to 0.7 mg/kg [11]. This conclusion was mainly based on studies reporting developmental toxicity in rats and concern about high consumption estimates in the population. As a result, the EFSA performed a refined exposure assessment for Ponceau 4R, using new data from the food industry (3 out of 31 food categories for which Ponceau 4R is authorized), analytical data submitted to the EFSA by the EU Member States (18 food categories), and food consumption data from the EFSA Comprehensive European Food Consumption Database. The report concluded that no consumption scenario exceeds the ADI; the highest consumption of Ponceau 4R was observed in toddlers and children—up to 0.51 mg/kg per day [70]. 

Research on this color’s toxicity is ongoing. The mutagenic potential of the color and its six photo-degradation products found in a sweet beverage were assessed [71]. This approach enables the estimation of the toxic potential arising from interactions of the color with other beverage ingredients, such as citric acid, fructose syrup, sucrose, and grape juice. Hence, the study concluded that toxicity should be evaluated for every beverage, not just the color [71]. An in vitro study assessing the interaction of Ponceau 4R with P-glycoprotein did not indicate any such interaction [31].

Recent evidence from animal studies regarding Ponceau 4R revealed a potential to alter the structure and thickness of the duodenal wall in rats receiving a mixture of sodium nitrite, monosodium glutamate, and Ponceau 4R for 16 weeks [72]. The doses of the additives were high (5 mg/kg for Ponceau 4R), but these findings may indicate a certain synergy in the toxicity of the additives used in meat products. Recently, a model for screening for potential alterations in cardiovascular functionality due to prenatal toxic insult was established using zebrafish [73]. In this model, Ponceau 4R affected the zebrafish pectoral fin swing behavior and increased the heart rate of juvenile fish by 32% [73]. This suggests the dye has developmental toxicity.

Furthermore, an in vitro study revealed the potential of Ponceau 4R to enhance the production of leukotriene B4 in blood neutrophils. This indicates a pro-inflammatory potential, particularly related to asthma. The effect was present at all tested concentrations, ranging from 10 to 100 μmol/L [55]. A recent clinical study examined suspected food color reactions in nineteen children orally administered 2.5 mg of a color [74]. In the case of Ponceau 4R, an allergic reaction was confirmed in only one child, manifesting as urticaria. However, the authors admit that reactions may manifest after ingesting a higher dose [74]. 

Both the EFSA and JECFA issued their evaluations more than a decade ago; hence, new assessments are warranted. 

### 2.6. E 127 ERYTHROSINUM NATRICUM, Erythrosine

Erythrosine as a red dye is authorized by the EFSA exclusively for cocktail and candied cherries and Bigarreaux cherries. Its safety was re-evaluated by the EFSA in 2011 and by the JECFA in 1990 and 2018. The main concern regarding Erythrosine is its potential ability to induce tumorigenesis in the thyroid gland of rodents, even though this effect is secondary to its effects on thyroid function and not related to any genotoxic activity [12,13]. 

However, a genotoxic effect of Erythrosine was observed in HepG2 cells exposed to 70 mg/L. The results showed decreased expression of two genes (*FEN1* and *REV1*) related to DNA base repair [30]. The toxicity was further examined on *Drosophila melanogaster*, where exposure to concentrations from 0.000005 mg/mL to 0.05 mg/mL decreased longevity. Furthermore, the exposure of HL-60 cells to the same dose range inhibited proliferation and did not induce further tumor cell growth, suggesting no cancerogenic effect [75]. These studies show potential harm to living cells in both in vitro and in vivo models at very low concentrations. 

Moreover, Erythrosine administered together with Tartrazine at a dose of 20 mg/kg of body weight showed testicular tissue damage after 23 days of exposure, as well as a disruption in the hormonal regulation of sex hormones [76]. These doses were very high, massively exceeding the ADI of Erythrosine and markedly exceeding that of Tartrazine. The same mixture of azo dyes was used to assess a potential memory impairment effect. Rats were exposed to a 50:50 mixture of Erythrosine and Tartrazine at concentrations of 2, 6, and 10 mg/kg of body weight for six weeks. Interestingly, while low doses enhanced nonspatial memory retention, higher concentrations led to impaired nonspatial memory retention as well as an increase in anxiety-like behavior. On the histopathological level, there was a dose-dependent effect on the increase in acetylcholinesterase activity in hippocampal tissues and up-regulation of oxidative stress markers, including TNFα. This study also showed a potential metabolic effect of these food colors, as groups exposed to 6 and 10 mg/kg of body weight experienced a decrease in body weight from the fourth week of treatment to the last week [77]. However, the study employed very high Erythrosine doses (all exceeding the ADI), while Tartrazine dosing was below the ADI in all doses. This makes the results difficult to interpret in the context of human exposure. Another study focusing on the metabolic effects of a Tartrazine and Erythrosine mixture showed higher glycemia in groups treated with very high doses of the dyes—a mixture of 20 and 40 mg/kg of body weight, respectively, for 21 days [78]. 

Higher levels of TNFα were also observed in a study focusing on kidney injury induced by the mixture of Erythrosine and Tartrazine. Rats were exposed to a 50:50 mixture in concentrations varying from 2.5 to 20 mg/kg of body weight. However, this study did not show any concentration-dependent effects on biochemical and gene expression markers. Caspase-9 and kidney injury molecule-1 were up-regulated in groups treated with low doses (2.5 and 5 mg/kg of body weight), while high-dose treatments (10 and 20 mg/kg of body weight) led to down-regulation of these markers. Serum urea concentrations were increased in groups treated with 5 and 20 mg/kg and decreased in groups treated with 10 mg/kg.

In contrast, serum creatinine levels significantly increased after 10 and 20 mg/kg exposure. While these biochemical markers did not show a dose-dependent effect, histopathological examination revealed hypertrophy of the glomeruli in relation to the size of the Bowman’s capsule in the groups treated with the two highest concentrations [79]. However, these concentrations were above the recommended ADIs; therefore, the results serve purely to conceptualize the potential combined toxicity.

Neuronal toxicity of Erythrosine alone was examined in chicken embryos incubated with low (0.05 mg/kg) and high (0.1 mg/kg) doses, the higher dose reflecting the current ADI limit. Toxicity was observed at the histopathological level, as shown by the higher occurrence of neuronal tube defects in groups treated with Erythrosine [80].

Notably, it has been shown that Erythrosine could also have beneficial effects. An in vitro study showed that exposure to Erythrosine could dose-dependently decrease lysozyme fibrillogenesis [81]. Additionally, Erythrosine, in combination with nano-TiO_2_ in photodynamic therapy, could lead to decreased survival of *Candida albicans*, where Erythrosine acts as an effective photosensitizer [82]. Similar results were reported in a study focusing on chitosan potentiation of antimicrobial photodynamic inactivation together with Erythrosine, where the combination of chitosan with Erythrosine promoted not only antimicrobial activity against *Candida albicans* but also against *Streptococcus mutans* and *Pseudomonas aeruginosa* [83].

Taken together, Erythrosine has a proven toxicity, which is reflected in a very low ADI and its limited use. Many recent toxicity studies have focused on 50:50 mixtures of Erythrosine and Tartrazine, which do not mimic the ADI levels, and many of them are from one laboratory and their results have not been replicated. While Erythrosine clearly shows a promising novel approach to treating infectious diseases, its toxic effects on cells must be considered while examining its potential benefits.

### 2.7. E 129 RUBOR ALLURA, Allura Red

Allura Red is not among the widely used food colors. Nevertheless, it can pose a potential health risk. It was evaluated by the EFSA in 2009, and the most recent assessment of its toxicity as a food color was conducted by the JECFA in 2016, which concluded that there was no need to revise the current ADI and that the estimated dietary exposures remained below this level [4]. 

Earlier results indicating genotoxicity [84] have not been confirmed by more extensive studies [85,86]. However, even at doses relevant to exposure in food, toxicity has been found. Allura Red caused histopathological and physiological aberrations in the liver and kidney in rats given the ADI dose for 4 weeks [45]. After 6 weeks of ADI doses, rats showed behavioral signs of spatial memory impairment and reduction in the number of glial cells, while high doses (10 times the ADI level) were shown to exert additional massive effects on learning and memory, with multiple structural changes in the prefrontal cortex [87]. Interestingly, this study also showed that taurine could prevent the neurotoxicity of both Allura Red doses [87].

This dye is associated with allergies, intolerances, and hypersensitivities. Neutrophils and reactive oxygen species (ROSs) play an essential role in their development. Allura Red causes a significant and dose-dependent inhibition of ROS production by PMNs (human polymorph nuclear leukocytes) [54]. Furthermore, Allura Red may also be one of the external triggers that promote the manifestation of autoimmune diseases. It caused rapid development of colitis in genetically predisposed mice (increased IL-23 signaling) via activation of CD4+ T cells, leading to apoptosis of intestinal epithelial cells [88,89]. 

Allura Red may affect extra- and intra-cellular activities via binding with proteins, including enzymes. It binds to human serum albumin [90], although this effect would likely be negligible at ADI levels. Allura Red and its metabolites also inhibit carbonic anhydrase activity [91,92], pepsin, and trypsin [93,94]; however, no effect on P-glycoprotein was found [31]. These effects may lead to toxicity or drug–drug interactions at high doses, but they are unlikely to be biologically relevant at ADI exposure. A study investigating the potential developmental toxicity of azo dyes using zebrafish embryos came to the same conclusion: a positive finding will only be apparent at concentrations higher than the average intake in the human population [35,95].

It is not surprising that high chronic doses of Allura Red are neurotoxic and nephrotoxic in rats. The administration of 200 mg of the color for 4 weeks led to increased levels of indicators of oxidative stress (malonaldehyde and glutathione peroxidase), which probably led to kidney and brain damage and changes in the levels of serotonin and GABA in several brain regions [96]. An increased level of urea or creatinine as an indicator of renal dysfunction was observed in the plasma of rats after administration of 50 mg/kg Allura Red for 10 and 40 days [97]. The same study pointed to probable toxicity in other tissues, manifested by increased levels of the serum aminotransferases (ASL, ALT), ALP, glucose, and serum total protein and decreased levels of erythrocytes and hemoglobin [97].

In summary, this dye requires continuous monitoring as some in vivo studies indicate detrimental effects even at ADI doses.

### 2.8. E 131 CERULEUM PROTECTUM, Patent Blue

The main concern regarding the use of Patent Blue is its potential to induce allergic reactions or anaphylaxis during surgery—the occurrence of anaphylactic reactions after perioperative intravenous administration is rare but well documented [98]. However, such issues are unrelated to using the dye as a food additive. The EFSA decreased the ADI in 2013 from 15 mg/kg to 5 mg/kg, but this was motivated by unwanted hematologic effects occurring after chronic intake of high doses [1,15]. A clinical study examining suspected food color reactions in children found no allergic reaction after an oral food challenge of 2.5 mg of Patent Blue in nine children [74]. While it is possible to substitute other colors with natural colors, blue will likely remain an issue, as it is hard to find a stable natural source that is usable in the food industry [99]. The EFSA evaluated Patent Blue in 2013 and the JECFA in 1982. Since then, there has been little new evidence, and the color seems to be safe at the current ADI limit.

### 2.9. E 132 INDIGOCARMINUM, Indigo Carmine

At the time of the EFSA’s re-evaluation, Indigo Carmine did not raise any concerns regarding developmental toxicity, genotoxic, or cytotoxicity [1,17]. The JECFA reached a similar conclusion in 2019 [13].

However, some recent in vitro studies indicate that Indigo Carmine can enter human fibroblast cells and exhibit cytotoxicity; for example, a reduction in cell proliferation by 60–80% has been found [100]. Another in vitro study using tumor human HL-60 cells showed some growth inhibition, and an in vivo experiment from the same report using Drosophila revealed a short healthy lifespan after 6 days of exposure [75]. Similarly, a report on the exposure of Tenebrio molitor larvae to Indigo Carmine indicated lowered body weight, but the concentration was very high—1 g/kg administered over 3 weeks [101]. These data are difficult to extrapolate to human exposure, and there is likely a very low risk when Indigo Carmine is used as a food color; however, its possible toxicity deserves further research. 

Interestingly, Indigo Carmine was shown to have some antioxidant activity [54,102]. However, in a study designed to test this effect, Indigo Carmine failed to prevent ischemia-reperfusion injury in a model of isolated liver and worsened the damage to the hepatocyte membranes [103]. 

Given the recent JECFA evaluation, it seems that this color is safe under the current ADI limit, set as 0-5 mg/kg of body weight by both the EFSA and JECFA.

### 2.10. E 133 CERULEUM NITENS, Brilliant Blue FCF 

Brilliant Blue was assessed by the EFSA in 2010 and the JECFA in 2017, and both authorities confirmed the same ADI [18,19].

Brilliant blue is known to have some cytotoxic and genotoxic potential [104], and new studies are addressing this concern. Genotoxicity was assessed by comet assay in human male germ cells, showing that only the highest concentrations of Brilliant Blue (200 and 500 μg/mL) induced DNA damage in sperm cells [40]. A comprehensive study using in vitro tests on the HL-60 tumor human cell line and in vivo experiments with Drosophila larvae confirmed the safety of the color at concentrations corresponding to the ADI but showed enhanced growth of the tumor cell line [75]. Interestingly, Brilliant Blue improved the longevity of the Drosophila larvae in the same study [75]. Hence, even these optimistic results do not rule out potential risks of regular exposure to the color. 

A stem-cell-based in vitro morphogenesis assay was employed to assess the developmental toxicity of Brilliant Blue. The results found a NOAEL of 400 μM and a LOAEL of 600 μM [105]. However, these levels may be higher than the typical population exposure. Therefore, the potential health risk during pregnancy cannot be assessed on the basis of these findings. The zebrafish model for screening of developmental toxicity for cardiovascular functionality (see also Ponceau 4R) showed that Brilliant Blue increased the heart rate of the fish by 10% [73], suggesting specific developmental issues related to the dye. 

Hematological and immune alterations were also assessed on the whole-organism level by exposing rats to oral administration of 1.2 mg/kg for 90 days [106]. Specifically, the results showed that exposure to Brilliant Blue reduced both white and red blood cell counts, hemoglobin content, and the number of platelets but increased the mean corpuscular volume of the red blood cells. Furthermore, the color reduced the total IgM and IgG levels and increased phagocytic activity [106]. Future studies shall determine the importance of these results for consumers’ safety.

There have also been positive reports indicating Brilliant Blue could be useful in clinical medicine or represent a promising molecule for new drug development. The color could safely replace gentian violet as a skin marker, as gentian violet has detrimental effects on the physiologic functions of vein grafts during cardiac bypass surgery [107]. Furthermore, Brilliant Blue seems to suppress the production of uncontrolled neutrophil extracellular traps under excessive inflammatory conditions [108]. Lastly, Brilliant Blue dose-dependently inhibited amyloid fibril formation of lysozymes [109]. These findings may lead to the development of new therapeutic approaches for inflammatory and neurodegenerative diseases.

### 2.11. E 142 VIRIDE S, Green S

Given the poor solubility of Green S, this color seems to be safe at the current ADI, which has remained unchanged since 1974. For this review, we did not find any clinical, preclinical, *or* in vitro studies relevant for evaluating its toxicity in humans. 

### 2.12. E 155 FUSCUM HT, Brown HT

Brown HT seems safe under the ADI set by the EFSA in 2010, but the panel raised a concern about potentially higher dietary intake of the dye in children. Therefore, the EFSA conducted a new exposure assessment and collected usage levels for 6 out of 37 food categories in which Brown HT is authorized and a limited number of food analytical results, with all found to be below the limit of detection or quantification. In one consumption scenario, at the 95th percentile in all populations, the elderly exceeded the ADI. However, such a situation is unlikely to occur in the population [110]. For this review, we did not find any recent clinical, preclinical, or in vitro studies relevant for evaluating the toxicity of Brown HT in humans. 

### 2.13. E 151 NIGRUM NITENS, Brilliant Black

Recently, the JECFA published a new assessment of the toxicity of Brilliant Black and retained the 1 mg ADI set in 1981 [24], while the Scientific Committee for Food (SCF, later transformed to the EFSA) still retains the higher 5 mg ADI level from 1984 [23]. Later, the EFSA performed a refined exposure assessment for Brilliant Black due to the concern raised in the Scientific Opinion about consumption exceeding the ADI in children younger than 10 years old. The EFSA collected usage levels for 11 out of 37 food categories in which the color is authorized. Furthermore, 4337 food analytical results were available, with most values being below the limit of detection or quantification. So far, Brilliant Black seems safe under the current ADI limits. The EFSA concluded that exceeding the ADI in any population under any consumer scenario is unlikely [111].

Interestingly, Brilliant Black recently showed promising antiviral activity against enterovirus 71, which causes hand, foot, and mouth disease [112]. It seems that sulfonated azo dyes exhibit a certain ability to block the entry of the virus into the cell. However, Brilliant Black showed the highest potential, which was confirmed in vivo [112]. This is not the first report of Brilliant Black as a potential pharmacological treatment option. It was shown to inhibit the affinity of A1 and A3 adenosine receptor agonists [113], but this line of research has so far not led to further drug development.

Considering the recent JECFA evaluation, it seems that this color is safe under the current ADI limits, set as 0–5 mg/kg of body weight by the EFSA and 0–1 mg/kg of body weight by the JECFA. 

### 2.14. E 180 LITHOLRUBINUM BK, Lithol Rubine BK

Lithol Rubine BK is a red dye that is generally used in cosmetics. It is also used in the food industry exclusively as a surface coating in cheese [114]. Toxicological studies were performed mainly in the late 1980s and early 1990s. The last re-evaluation by the EFSA was in 2010, and the last evaluation by the JECFA was in 1987. However, because of a lack of data in 1987, the JECFA could not establish an ADI. In contrast, the EU Scientific Committee for Food established an ADI of 0–1.5 mg/kg of body weight/day [25]. This ADI was determined on the basis of a NOAEL of 100 mg/kg of body weight/day from a long-term rat study established by the BIBRA toxicity profile of Lithol Rubine BK. In this study, rats exposed to 1 g/kg of body weight for 30 days had slightly reduced growth. There was also an increase in kidney weight, and a histopathological examination showed kidney damage. However, these effects were reversible over a two-week recovery period [115]. Chronic toxicity was also examined by a two-year feeding study in dogs. Six dogs (3 per sex) were administered 0.015, 0.1, and 1.0% of Lithol Rubine BK solution (about 250 mg/kg of body weight per day). A microscopic evaluation of various tissues showed no significant effect of the highest concentration [115]. 

The mutagenic potential was examined in vitro in mouse lymphoma L5178Y cells, and there were no effects suggesting the genotoxicity of Lithol Rubine BK [25]. On the basis of the limited data and the fact that the highest level of anticipated exposure is 1700-fold lower than the identified effect level of 100 mg/kg of body weight per day in female rats, the EFSA concluded that the existing ADI of 0–1.5 mg/kg should be withdrawn and considers it unlikely that there are any significant safety concerns for humans from the current, single-use authorization of Lithol Rubine BK in edible cheese rinds [25]. 

Interestingly, several azo dyes, including Lithol Rubine BK, were examined as potential candidates for the treatment of glioblastoma because of their antiproliferative effects. Lithol Rubine BK induced a concentration-dependent cytotoxic effect during a 3-day incubation with glioblastoma cells. The color caused an 8% to 51% increase in cellular deaths in concentrations of 2 μM to 64 μM, respectively [116]. The antiproliferative effect of Lithol Rubine BK on glioblastoma cells contrasts with previous toxicity studies. This fact may be attributed to the limited absorption of Lithol Rubine BK from the gastrointestinal system [117]. However, further assessment of Lithol Rubine BK as a potential antiproliferative treatment is necessary. 

## 3. Conclusions

We reviewed new lines of evidence regarding the azo dyes used as food colors. The issues regarding their toxicity and real population exposures are regularly reviewed by relevant food safety agencies such as the EFSA and JECFA. The food categories in which the colors are applied can be found in the European database [118] and the US Food and Drug Administration database [119]. The occurrence of food colors in specific foods is also regularly reviewed [120,121]. The current ADI limits set by the safety agencies are mostly in agreement, and they seem safe. In some cases, a new mechanism of action has been revealed, paving the road for the pharmacological repurposing of these compounds. Recently, there has been a debate on using functional food additives that may improve human health instead of those that are potentially harmful. The primary notion is to use exclusively nontoxic, plant-based food additives, which are safe from both a health and environmental point of view [122,123,124]. 

## Figures and Tables

**Table 1:** Glossary of commonly used terms.

Name	Acronym	Definition
Acceptable daily intake	ADI	An estimate of the amount of a substance in food or drinking water that can be consumed daily over a lifetime without presenting an appreciable risk to health. It is usually expressed as milligrams of the substance per kilogram of body weight per day.
No observed adverse effect level	NOAEL	The greatest concentration or amount of a substance at which no detectable adverse effects occur in an exposed population.
Lowest observed adverse effect level	LOAEL	The lowest level of a substance that has been observed to cause harm in an exposed population.
Scientific opinion		A document published by the EFSA. It includes risk assessments of general scientific issues; evaluations of applications for authorizations of products, substances, or claims; or evaluations of risk assessments.
Exposure assessment		A document published by the EFSA. It includes thorough evaluations of who or what have been exposed to hazards and quantifications of the amounts involved.
Evaluation of certain food additives		Xth Report of the Joint FAO/WHO Expert Committee on Food Additives, a document published by the WHO similar to the Scientific Opinion of the EFSA.

**Table 2:** Description of colors and their regulatory evaluations.

Code	Name	Description	ADI (EFSA)	EFSA Evaluation	ADI (JECFA)	JECFA Evaluation
E 104	FLAVUM QUINOLINI Quinoline Yellow	Yellow water-soluble anionic quinophthalone dye	0–0.5 mg/kg	2009 [3]	0–3 mg/kg	2016 [4]
E 110	FLAVUM ORANGEATUM Sunset Yellow	Orange water-soluble anionic monoazo dye	0–4 mg/kg	2014 [5]	0–4 mg/kg	2011 [6]
E 122	AZORUBINUM Azorubine, also known as Carmoisine	Red water-soluble anionic monoazo dye	0–4 mg/kg	2009 [7]	0–4 mg/kg	1983 [8]
E 123	AMARANTHUM Amaranth	Red water-soluble anionic monoazo dye	0–0.15 mg/kg	2010 [9]	0–0.5 mg/kg	1984 [10]
E 124	RUBOR PONCEAU Ponceau 4R	Red water-soluble anionic monoazo dye	0–0.7 mg/kg	2009 [11]	0–4 mg/kg	2011 [6]
E 127	ERYTHROSINUM NATRICUM Erythrosine	Red xanthene dye	0–0.1 mg/kg	2011 [12]	0–0.1 mg/kg	2018 [13]
E 129	RUBOR ALLURA Allura Red	Red water-soluble anionic monoazo dye	0–7 mg/kg	2009 [14]	0–7 mg/kg	2016 [4]
E 131	CERULEUM PROTECTUM Patent Blue	Blue water-soluble anionic triphenylmethane dye	0–5 mg/kg	2013 [15]	no ADI	1982 [16]
E 132	INDIGOCARMINUM Indigo Carmine	Blue water-soluble anionic pyrrole-based dye	0–5 mg/kg	2014 [17]	0–5 mg/kg	2019 [13]
E 133	CERULEUM NITENS Brilliant Blue FCF	Blue water-soluble anionic triphenylmethane dye	0–6 mg/kg	2010 [18]	0–6 mg/kg	2017 [19]
E 142	VIRIDE S Green S	Green water-soluble anionic triarylmethane dye	0–5 mg/kg	2010 [20]	no ADI	1974 [21]
E 155	FUSCUM HT Brown HT	Brown water-soluble anionic diazo dye	0–1.5 mg/kg	2010 [22]	0–1.5 mg/kg	1984 [10]
E 151	NIGRUM NITENS Brilliant Black	Black water-soluble anionic diazo dye	0–5 mg/kg	2010 [23]	0–1 mg/kg	2019 [24]
E 180	LITHOLRUBINUM BK Lithol Rubine BK	Red water-soluble monoazo dye	0–1.5 mg/kg, withdrawn	2010 [25]	not assessed	

ADI is expressed as mg/kg of body weight per day.

## Data Availability

Not applicable.

## References

[1] Amchova P., Kotolova H., Ruda-Kucerova J. (2015). Health Safety Issues of Synthetic Food Colorants. Regul. Toxicol. Pharmacol..

[2] Oplatowska-Stachowiak M., Elliott C.T. (2017). Food Colors: Existing and Emerging Food Safety Concerns. Crit. Rev. Food Sci. Nutr..

[3] EFSA Panel on Food Additives and Nutrient Sources Added to Food (2009). Scientific Opinion on the Re-Evaluation of Quinoline Yellow (E 104) as a Food Additive. EFSA J..

[4] World Health Organization (2016). Evaluation of Certain Food Additives: Eighty-Second Report of the Joint FAO.

[5] EFSA Panel on Food Additives and Nutrient Sources added to Food (ANS) (2014). Reconsideration of the Temporary ADI and Refined Exposure Assessment for Sunset Yellow FCF (E 110). EFSA J..

[6] World Health Organization (2011). Evaluation of Certain Food Additives and Contaminants: Seventy-Fourth [74th] Report of the Joint FAO/WHO Expert Committee on Food Additives.

[7] EFSA Panel on Food Additives and Nutrient Sources Added to Food (2009). Scientific Opinion on the Re-Evaluation of Azorubine/Carmoisine (E 122) as a Food Additive. EFSA J..

[8] Joint FAO/WHO Expert Committee on Food Additives (JECFA) (1983). Evaluation of Certain Food Additives and Contaminants: Twenty-Seventh Report of the Joint FAO.

[9] EFSA Panel on Food Additives and Nutrient Sources added to Food (ANS) (2010). Scientific Opinion on the Re-Evaluation of Amaranth (E 123) as a Food Additive. EFSA J..

[10] Joint FAO/WHO Expert Committee on Food Additives (JECFA) (1984). Evaluation of Certain Food Additives and Contaminants: Twenty-Eighth Report of the Joint FAO.

[11] EFSA Panel on Food Additives and Nutrient Sources Added to Food (2009). Scientific Opinion on the Re-Evaluation of Ponceau 4R (E 124) as a Food Additive. EFSA J..

[12] EFSA Panel on Food Additives and Nutrient Sources added to Food (ANS) (2011). Scientific Opinion on the Re-Evaluation of Erythrosine (E 127) as a Food Additive. EFSA J..

[13] World Health Organization (2019). Evaluation of Certain Food Additives: Eighty-Sixth Report of the Joint FAO/WHO Expert Committee on Food Additives.

[14] EFSA Panel on Food Additives and Nutrient Sources Added to Food (2009). Scientific Opinion on the Re-Evaluation of Allura Red AC (E 129) as a Food Additive. EFSA J..

[15] EFSA Panel on Food Additives and Nutrient Sources added to Food (ANS) (2013). Scientific Opinion on the Re-Evaluation of Patent Blue V (E 131) as a Food Additive. EFSA J..

[16] World Health Organization (1982). Evaluation of Certain Food Additives and Contaminants: Twenty Sixth Report of the Joint FAO/WHO Expert Comittee on Food Additives.

[17] EFSA Panel on Food additives and Nutrient Sources added to Food (ANS) (2014). Scientific Opinion on the Re-Evaluation of Indigo Carmine (E 132) as a Food Additive. EFSA J..

[18] EFSA Panel on Food Additives and Nutrient Sources added to Food (ANS) (2010). Scientific Opinion on the Re-Evaluation of Brilliant Blue FCF (E 133) as a Food Additive. EFSA J..

[19] World Health Organization (2017). Evaluation of Certain Food Additives: Eighty-Fourth Report of the Joint FAO.

[20] EFSA Panel on Food Additives and Nutrient Sources added to Food (ANS) (2010). Scientific Opinion on the Re-Evaluation of Green S (E 142) as a Food Additive. EFSA J..

[21] World Health Organization (1974). Eighteenth Report of the Joint FAO/WHO Expert Committee on Food Additives.

[22] EFSA Panel on Food Additives and Nutrient Sources (ANS) (2010). Scientific Opinion on the Re-Evaluation of Brown HT (E 155) as a Food Additive. EFSA J..

[23] EFSA Panel on Food Additives and Nutrient Sources added to Food (ANS) (2010). Scientific Opinion on the Re-Evaluation of Brilliant Black BN (E 151) as a Food Additive. EFSA J..

[24] World Health Organization (2019). Evaluation of Certain Food Additives: Eighty-Seventh Report of the Joint FAO.

[25] EFSA Panel on Food Additives and Nutrient Sources added to Food (ANS) (2010). Scientific Opinion on the Re-Evaluation of Litholrubine BK (E 180) as a Food Additive. EFSA J..

[26] European Food Safety Authority (2015). Refined Exposure Assessment for Quinoline Yellow (E 104). EFSA J..

[27] Khan M.S., Bhatt S., Tabrez S., Rehman M.T., Alokail M.S., AlAjmi M.F. (2020). Quinoline Yellow (Food Additive) Induced Conformational Changes in Lysozyme: A Spectroscopic, Docking and Simulation Studies of Dye-Protein Interactions. Prep. Biochem. Biotechnol..

[28] Khan M.S., Rehman M.T., Bhat S.A., Tabrez S., Hussain A., Husain F.M., AlAjmi M.F., Alamery S.F., Sumbul S. (2019). Food Additive Dye (Quinoline Yellow) Promotes Unfolding and Aggregation of Myoglobin: A Spectroscopic and Molecular Docking Analysis. Spectrochim. Acta Part A Mol. Biomol. Spectrosc..

[29] Tarnow P., Zordick C., Bottke A., Fischer B., Kühne F., Tralau T., Luch A. (2020). Characterization of Quinoline Yellow Dyes As Transient Aryl Hydrocarbon Receptor Agonists. Chem. Res. Toxicol..

[30] Chequer F.M., Venancio V.P., Almeida M.R., Aissa A.F., Bianchi M.L.P., Antunes L.M. (2017). Erythrosine B and Quinoline Yellow Dyes Regulate DNA Repair Gene Expression in Human HepG2 Cells. Toxicol. Ind. Health.

[31] Staples J.W., Stine J.M., Mäki-Lohiluoma E., Steed E., George K.M., Thompson C.M., Woodahl E.L. (2020). Food Dyes as P-Glycoprotein Modulators. Food Chem. Toxicol..

[32] Rovina K., Siddiquee S., Shaarani S.M. (2017). Highly Sensitive Electrochemical Determination of Sunset Yellow in Commercial Food Products Based on CHIT/GO/MWCNTs/AuNPs/GCE. Food Control.

[33] Supramaniam G., Warner J.O. (1986). Artificial Food Additive Intolerance in Patients with Angio-Oedema and Urticaria. Lancet.

[34] Van Bever H.P., Docx M., Stevens W.J. (1989). Food and Food Additives in Severe Atopic Dermatitis. Allergy.

[35] Jiang L.-L., Li K., Yan D.-L., Yang M.-F., Ma L., Xie L.-Z. (2020). Toxicity Assessment of 4 Azo Dyes in Zebrafish Embryos. Int. J. Toxicol..

[36] Joshi V., Pancharatna K. (2019). Food Colorant Sunset Yellow (E110) Intervenes Developmental Profile of Zebrafish (*Danio rerio*). J. Appl. Toxicol..

[37] Colakoglu F., Selcuk M.L. (2021). The Embryotoxic Effects of in Ovo Administered Sunset Yellow FCF in Chick Embryos. Vet. Sci..

[38] Çolakoğlu F., Selçuk M.L. (2020). Effects of Sunset Yellow FCF on Immune System Organs during Different Chicken Embryonic Periods. J. Vet. Res..

[39] Bastaki M., Mendes O.R., Bauter M.R., Taylor S.V. (2019). Assessment of FD&C Yellow No. 6 (Sunset Yellow FCF) Effects on Sperm Count, Motility and Viability in the Rat in a 28-Day Toxicity Study. Regul. Toxicol. Pharmacol..

[40] Pandir D. (2016). DNA Damage in Human Germ Cell Exposed to the Some Food Additives In Vitro. Cytotechnology.

[41] Dwivedi K., Kumar G. (2015). Genetic Damage Induced by a Food Coloring Dye (Sunset Yellow) on Meristematic Cells of *Brassica campestris* L.. J. Environ. Public Health.

[42] Haverić A., Haverić S., Hadžić M., Lojo-Kadrić N., Ibrulj S. (2018). Genotoxicity and Cytotoxicity Analysis of Curcumin and Sunset Yellow in Human Lymphocyte Culture. Cell. Mol. Biol..

[43] Ali M.Y., Hassan G.M., Hassan A.M.S., Mohamed Z.A., Ramadan M.F. (2020). In Vivo Genotoxicity Assessment of Sunset Yellow and Sodium Benzoate in Female Rats. Drug Chem. Toxicol..

[44] Saxena B., Sharma S. (2015). Food Color Induced Hepatotoxicity in Swiss Albino Rats, Rattus Norvegicus. Toxicol. Int..

[45] Khayyat L.I., Essawy A.E., Sorour J.M., Soffar A. (2018). Sunset Yellow and Allura Red Modulate Bcl2 and COX2 Expression Levels and Confer Oxidative Stress-Mediated Renal and Hepatic Toxicity in Male Rats. PeerJ.

[46] Kong X., Wang X., Qin Y., Han J. (2021). Effects of Sunset Yellow on Proliferation and Differentiation of Intestinal Epithelial Cells in Murine Intestinal Organoids. J. Appl. Toxicol..

[47] Ali M.Y., Hassan A.M.S., Mohamed Z.A., Ramadan M.F. (2019). Effect of Food Colorants and Additives on the Hematological and Histological Characteristics of Albino Rats. Toxicol. Environ. Health Sci..

[48] Elbanna K., Sarhan O.M., Khider M., Elmogy M., Abulreesh H.H., Shaaban M.R. (2017). Microbiological, Histological, and Biochemical Evidence for the Adverse Effects of Food Azo Dyes on Rats. J. Food Drug Anal..

[49] European Food Safety Authority (2015). Refined Exposure Assessment for Azorubine/Carmoisine (E 122). EFSA J..

[50] Khan I.S., Ali S., Dar K.B., Murtaza M., Ali M.N., Ganie S.A., Dar S.A. (2022). Toxicological Analysis of Synthetic Dye Orange Red on Expression of NFκB-Mediated Inflammatory Markers in Wistar Rats. Drug Chem. Toxicol..

[51] Al Reza M.S., Hasan M.M., Kamruzzaman M., Hossain M.I., Zubair M.A., Bari L., Abedin M.Z., Reza M.A., Khalid-Bin-Ferdaus K.M., Haque K.M.F. (2019). Study of a Common Azo Food Dye in Mice Model: Toxicity Reports and Its Relation to Carcinogenicity. Food Sci. Nutr..

[52] Panachiyil G.M., Babu T., Sebastian J., Doddaiah N. (2019). A Pediatric Case Report of Fixed Drug Eruption Related to Carmoisine Colorant Present in Paracetamol Syrup. Indian J. Pharmacol..

[53] Gülseren D., Hapa A., Ersoy-Evans S., Elçin G., Karaduman A. (2017). Is There a Role of Food Additives in Recurrent Aphthous Stomatitis? A Prospective Study with Patch Testing. Int. J. Dermatol..

[54] Al-Shammari E., Epuru S., Bano R., Adnan M., Khan S. (2016). Allura Red, Carmoisine and Indigo Carmine Inhibit Reactive Oxygen Species Production by Human Polymorphonuclear Leukocytesin Vitro. Egypt. Acad. J. Biol. Sci. C Physiol. Mol. Biol..

[55] Leo L., Loong C., Ho X.L., Raman M.F.B., Suan M.Y.T., Loke W.M. (2018). Occurrence of Azo Food Dyes and Their Effects on Cellular Inflammatory Responses. Nutrition.

[56] Amin K.A., Hameid H.A., Abd Elsttar A.H. (2010). Effect of Food Azo Dyes Tartrazine and Carmoisine on Biochemical Parameters Related to Renal, Hepatic Function and Oxidative Stress Biomarkers in Young Male Rats. Food Chem. Toxicol..

[57] Raposa B., Pónusz R., Gerencsér G., Budán F., Gyöngyi Z., Tibold A., Hegyi D., Kiss I., Koller Á., Varjas T. (2016). Food Additives: Sodium Benzoate, Potassium Sorbate, Azorubine, and Tartrazine Modify the Expression of NFκB, GADD45α, and MAPK8 Genes. Physiol. Int..

[58] Montaser M., Abiya R.A., Afifi M., Saddick S., Allogmani A.S., Almaghrabi O.A. Effect of Natural and Synthetic Food Colorants on Spermatogenesis and the Expression of Its Controlling Genes. Proceedings of the 3rd International Conference, Veterinary Medicine In-Between Health and Economy (VMHE).

[59] Kiziltan T., Baran A., Kankaynar M., Şenol O., Sulukan E., Yildirim S., Ceyhun S.B. (2022). Effects of the Food Colorant Carmoisine on Zebrafish Embryos at a Wide Range of Concentrations. Arch. Toxicol..

[60] Khan I.S., Ali M.N., Hamid R., Ganie S.A. (2020). Genotoxic Effect of Two Commonly Used Food Dyes Metanil Yellow and Carmoisine Using *Allium cepa* L. as Indicator. Toxicol. Rep..

[61] Basu A., Suresh Kumar G. (2017). Interaction and Inhibitory Influence of the Azo Dye Carmoisine on Lysozyme Amyloid Fibrillogenesis. Mol. Biosyst..

[62] Radomski J.L., Mellinger T.J. (1962). The Absorption, Fate and Excretion in Rats of the Water-Soluble Azo Dyes, FD&C Red No. 2, FD&C Red No. 4, and FD&C Yellow No. 6. J. Pharmacol. Exp. Ther..

[63] Phillips J.C., Bex C.S., Mendis D., Walters D.G., Gaunt I.F. (1987). Metabolic Disposition of 14C-Labelled Amaranth in the Rat, Mouse and Guinea-Pig. Food Chem. Toxicol..

[64] Ruddick J.A., Craig J., Stavric B., Willes R.F., Collins B. (1979). Uptake, Distribution and Metabolism of [14C] Amaranth in the Female Rat. Food Cosmet. Toxicol..

[65] Ruddick J.A., Craig J., Collins B. (1978). Pharmacokinetic and Transplacental Study of [C-14] Amaranth in Rat. Teratology.

[66] Ruddick J.A., Willes R.F., Stavric B., Munro I. (1977). Pharmacokinetics and Distribution of (C-14) Amaranth in Rat. Teratology.

[67] Jabeen H.S., ur Rahman S., Mahmood S., Anwer S. (2013). Genotoxicity Assessment of Amaranth and Allura Red Using Saccharomyces Cerevisiae. Bull. Environ. Contam. Toxicol..

[68] European Food Safety Authority (2013). Refined Exposure Assessment for Amaranth (E 123). EFSA J..

[69] Basu A., Kumar G.S. (2015). Interaction of Toxic Azo Dyes with Heme Protein: Biophysical Insights into the Binding Aspect of the Food Additive Amaranth with Human Hemoglobin. J. Hazard. Mater..

[70] European Food Safety Authority (2015). Refined Exposure Assessment for Ponceau 4R (E 124). EFSA J..

[71] Yamjala K., Nainar M.S., Kumar Varma S., Ambore N. (2016). Separation, Identification and Mutagenic Assessment of the Photodegradation Products of Ponceau 4R (E124) in a Beverage. Anal. Methods.

[72] Grigorenko A., Yeroshenko G., Shevchenko K., Lisachenko O., Perederii N. (2021). REMODELING OF THE RAT DUODENAL WALL UNDER THE EFFECT OF COMPLEX FOOD ADDITIVES OF MONOSODIUM GLUTAMATE, SODIUM NITRITE AND PONCEAU 4R. Georgian Med. News.

[73] Wang Y.-F., Chen I.-W., Subendran S., Kang C.-W., Panigrahi B., Fu T.-F., Chen C.-Y. (2020). Edible Additive Effects on Zebrafish Cardiovascular Functionality with Hydrodynamic Assessment. Sci. Rep..

[74] Lemoine A., Pauliat-Desbordes S., Challier P., Tounian P. (2020). Adverse Reactions to Food Additives in Children: A Retrospective Study and a Prospective Survey. Arch. Pédiatrie.

[75] Merinas-Amo R., Martínez-Jurado M., Jurado-Güeto S., Alonso-Moraga Á., Merinas-Amo T. (2019). Biological Effects of Food Coloring in In Vivo and In Vitro Model Systems. Foods.

[76] Wopara I., Modo E.U., Mobisson S.K., Olusegun G.A., Umoren E.B., Orji B.O., Mounmbegna P.E., Ujunwa S.O. (2021). Synthetic Food Dyes Cause Testicular Damage via Up-Regulation of pro-Inflammatory Cytokines and down-Regulation of FSH-R and TESK-1 Gene Expression. JBRA Assist. Reprod..

[77] Wopara I., Modo E.U., Adebayo O.G., Mobisson S.K., Nwigwe J.O., Ogbu P.I., Nwankwo V.U., Ejeawa C.U. (2021). Anxiogenic and Memory Impairment Effect of Food Color Exposure: Upregulation of Oxido-Neuroinflammatory Markers and Acetyl-Cholinestrase Activity in the Prefrontal Cortex and Hippocampus. Heliyon.

[78] Iheanyichukwu W., Uwaezuoke C.A., Amanda I. (2019). The Effect of Some Synthetic Food Colorants on Selected Biochemical Indices of Male Wistar Rats. Eur. J. Nutr. Food Saf..

[79] Iheanyichukwu W., Adegoke A.O., Adebayo O.G., Emmanuel U M., Egelege A.P., Gona J.T., Orluwene F.M. (2021). Combine Colorants of Tartrazine and Erythrosine Induce Kidney Injury: Involvement of TNF-α Gene, Caspase-9 and KIM-1 Gene Expression and Kidney Functions Indices. Toxicol. Mech. Methods.

[80] Ovalioglu A.O., Ovalioglu T.C., Arslan S., Canaz G., Aydin A.E., Sar M., Emel E. (2020). Effects of Erythrosine on Neural Tube Development in Early Chicken Embryos. World Neurosurg..

[81] Kuo C.-T., Chen Y.-L., Hsu W.-T., How S.-C., Cheng Y.-H., Hsueh S.-S., Liu H.-S., Lin T.-H., Wu J.W., Wang S.S.-S. (2017). Investigating the Effects of Erythrosine B on Amyloid Fibril Formation Derived from Lysozyme. Int. J. Biol. Macromol..

[82] Teerakapong A., Damrongrungruang T., Sattayut S., Morales N.P., Sangpanya A., Tanapoomchai M. (2017). Fungicidal Effect of Combined Nano TiO2 with Erythrosine for Mediated Photodynamic Therapy on Candida Albicans: An In Vitro Study. Lasers Dent. Sci..

[83] Chen C.-P., Chen C.-T., Tsai T. (2012). Chitosan Nanoparticles for Antimicrobial Photodynamic Inactivation: Characterization and In Vitro Investigation. Photochem. Photobiol..

[84] Tsuda S., Murakami M., Matsusaka N., Kano K., Taniguchi K., Sasaki Y.F. (2001). DNA Damage Induced by Red Food Dyes Orally Administered to Pregnant and Male Mice. Toxicol. Sci..

[85] Bastaki M., Farrell T., Bhusari S., Pant K., Kulkarni R. (2017). Lack of Genotoxicity In Vivo for Food Color Additive Allura Red AC. Food Chem. Toxicol..

[86] Honma M. (2015). Evaluation of the In Vivo Genotoxicity of Allura Red AC (Food Red No. 40). Food Chem. Toxicol..

[87] Noorafshan A., Hashemi M., Karbalay-Doust S., Karimi F. (2018). High Dose Allura Red, Rather than the ADI Dose, Induces Structural and Behavioral Changes in the Medial Prefrontal Cortex of Rats and Taurine Can Protect It. Acta Histochem..

[88] Chen L., He Z., Reis B.S., Gelles J.D., Chipuk J.E., Ting A.T., Spicer J.A., Trapani J.A., Furtado G.C., Lira S.A. (2022). IFN-Γ+ Cytotoxic CD4+ T Lymphocytes Are Involved in the Pathogenesis of Colitis Induced by IL-23 and the Food Colorant Red 40. Cell. Mol. Immunol..

[89] He Z., Chen L., Catalan-Dibene J., Bongers G., Faith J.J., Suebsuwong C., DeVita R.J., Shen Z., Fox J.G., Lafaille J.J. (2021). Food Colorants Metabolized by Commensal Bacteria Promote Colitis in Mice with Dysregulated Expression of Interleukin-23. Cell Metab..

[90] Wu D., Yan J., Wang J., Wang Q., Li H. (2015). Characterisation of Interaction between Food Colourant Allura Red AC and Human Serum Albumin: Multispectroscopic Analyses and Docking Simulations. Food Chem..

[91] Esmaeili S., Ashrafi-Kooshk M.R., Khaledian K., Adibi H., Rouhani S., Khodarahmi R. (2016). Degradation Products of the Artificial Azo Dye, Allura Red, Inhibit Esterase Activity of Carbonic Anhydrase II: A Basic In Vitro Study on the Food Safety of the Colorant in Terms of Enzyme Inhibition. Food Chem..

[92] Khodarahmi R., Kooshk M.R.A., Khaledian K. (2015). Allura Red, the Artificial Azo Dye, Inhibits Esterase Activity of Carbonic Anhydrase II: A Preliminary Study on the Food Safety in Term of Enzyme Inhibition. J. Rep. Pharm. Sci..

[93] Balaei F., Ansari M., Farhadian N., Moradi S., Shahlaei M. (2021). Interactions and Effects of Food Additive Dye Allura Red on Pepsin Structure and Protease Activity; Experimental and Computational Supports. Res. Pharm. Sci..

[94] Xiao Q., Liang J., Luo H., Li H., Yang J., Huang S. (2020). Investigations of Conformational Structures and Activities of Trypsin and Pepsin Affected by Food Colourant Allura Red. J. Mol. Liq..

[95] Doell D.L., Folmer D.E., Lee H.S., Butts K.M., Carberry S.E. (2016). Exposure Estimate for FD&C Colour Additives for the US Population. Food Addit. Contam. Part A.

[96] Bawazir A.E. (2016). Effects of Food Colour Allura Red (No. 129) on Some Neurotransmitter, Antioxidant Functions and Bioelement Contents of Kidney and Brain Tissues in Male Albino Rats. Life Sci. J..

[97] Alsolami M.A. (2017). Effect of a Food Additive on Certain Haematological and Biochemical Parameters in Male Albino Rat. Int. J. Zool. Res..

[98] Perenyei M., Barber Z.E., Gibson J., Hemington-Gorse S., Dobbs T.D. (2021). Anaphylactic Reaction Rates to Blue Dyes Used for Sentinel Lymph Node Mapping: Systematic Review and Meta-Analysis. Ann. Surg..

[99] Landim Neves M.I., Silva E.K., Meireles M.A.A. (2021). Natural Blue Food Colorants: Consumer Acceptance, Current Alternatives, Trends, Challenges, and Future Strategies. Trends Food Sci. Technol..

[100] Pasdaran A., Azarpira N., Heidari R., Nourinejad S., Zare M., Hamedi A. (2022). Effects of Some Cosmetic Dyes and Pigments on the Proliferation of Human Foreskin Fibroblasts and Cellular Oxidative Stress; Potential Cytotoxicity of Chlorophyllin and Indigo Carmine on Fibroblasts. J. Cosmet. Dermatol..

[101] Martynov V.O., Brygadyrenko V.V. (2018). The Influence of the Synthetic Food Colourings Tartrazine, Allura Red and Indigo Carmine on the Body Weight of *Tenebrio molitor* (Coleoptera, Tenebrionidae) Larvae. Regul. Mech. Biosyst..

[102] Farias-Silva E., Cola M., Calvo T.R., Barbastefano V., Ferreira A.L., Michelatto D.D.P., de Almeida A.C.A., Hiruma-Lima C.A., Vilegas W., Brito A.R.M.S. (2007). Antioxidant Activity of Indigo and Its Preventive Effect against Ethanol-Induced DNA Damage in Rat Gastric Mucosa. Planta Med..

[103] Rancan E.A., Frota E.I., de Freitas T.M.N., Jordani M.C., Évora P.R.B., Castro-e-Silva O. (2020). Evaluation of Indigo Carmine on Hepatic Ischemia and Reperfusion Injury. Acta Cir. Bras..

[104] Kus E., Eroglu H.E. (2015). Genotoxic and Cytotoxic Effects of Sunset Yellow and Brilliant Blue, Colorant Food Additives, on Human Blood Lymphocytes. Pak. J. Pharm. Sci..

[105] Yuan C.J., Marikawa Y. (2017). Developmental Toxicity Assessment of Common Excipients Using a Stem Cell-Based In Vitro Morphogenesis Model. Food Chem. Toxicol..

[106] Motwadie M.E., Hashem M.M., Abo-EL-Sooud K., Abd-Elhakim Y.M., El-Metwally A.E., Ali H.A. (2021). Modulation of Immune Functions, Inflammatory Response, and Cytokine Production Following Long-Term Oral Exposure to Three Food Additives; Thiabendazole, Monosodium Glutamate, and Brilliant Blue in Rats. Int. Immunopharmacol..

[107] Hocking K.M., Luo W., Li F.D., Komalavilas P., Brophy C., Cheung-Flynn J. (2016). Brilliant Blue FCF Is a Nontoxic Dye for Saphenous Vein Graft Marking That Abrogates Response to Injury. J. Vasc. Surg..

[108] Sofoluwe A., Bacchetta M., Badaoui M., Kwak B.R., Chanson M. (2019). ATP Amplifies NADPH-Dependent and -Independent Neutrophil Extracellular Trap Formation. Sci. Rep..

[109] Chen Y.-H., Tseng C.-P., How S.-C., Lo C.-H., Chou W.-L., Wang S.S.-S. (2016). Amyloid Fibrillogenesis of Lysozyme Is Suppressed by a Food Additive Brilliant Blue FCF. Colloids Surf. B Biointerfaces.

[110] European Food Safety Authority (2014). Refined Exposure Assessment of Brown HT (E 155). EFSA J..

[111] European Food Safety Authority (2015). Refined Exposure Assessment for Brilliant Black BN (E 151). EFSA J..

[112] Meng T., Jia Q., Wong S.-M., Chua K.-B. (2019). In Vitro and In Vivo Inhibition of the Infectivity of Human Enterovirus 71 by a Sulfonated Food Azo Dye, Brilliant Black BN. J. Virol..

[113] May L.T., Briddon S.J., Hill S.J. (2010). Antagonist Selective Modulation of Adenosine A1 and A3 Receptor Pharmacology by the Food Dye Brilliant Black BN: Evidence for Allosteric Interactions. Mol. Pharmacol..

[114] Mota I.G.C., Neves R.A.M.D., Nascimento S.S.D.C., Maciel B.L.L., Morais A.H.D.A., Passos T.S. (2023). Artificial Dyes: Health Risks and the Need for Revision of International Regulations. Food Rev. Int..

[115] BIBRA (British Industrial Biological Research Association) (1993). Toxicity Profile: Lithol Rubine BK.

[116] Sevastre A.-S., Baloi C., Alexandru O., Tataranu L.G., Popescu O.S., Dricu A. (2023). The Effect of Azo-Dyes on Glioblastoma Cells In Vitro. Saudi J. Biol. Sci..

[117] Leist K.H. (1982). Subacute Toxicity Studies of Selected Organic Colorants. Ecotoxicol. Environ. Saf..

[118] Database–European Commission. https://food.ec.europa.eu/safety/food-improvement-agents/additives/database_en.

[119] U.S. Food & Drug Administration (2024). Food Additive Listings.

[120] Pereira H., Deuchande T., Fundo J.F., Leal T., Pintado M.E., Amaro A.L. (2024). Painting the Picture of Food Colouring Agents: Near-Ubiquitous Molecules of Everyday Life—A Review. Trends Food Sci. Technol..

[121] Lehto S., Buchweitz M., Klimm A., Straßburger R., Bechtold C., Ulberth F. (2017). Comparison of Food Colour Regulations in the EU and the US: A Review of Current Provisions. Food Addit. Contam. Part A.

[122] Bashir I., Pandey V.K., Dar A.H., Dash K.K., Shams R., Mir S.A., Fayaz U., Khan S.A., Singh R., Zahoor I. (2024). Exploring Sources, Extraction Techniques and Food Applications: A Review on Biocolors as next-Generation Colorants. Phytochem. Rev..

[123] Zang E., Jiang L., Cui H., Li X., Yan Y., Liu Q., Chen Z., Li M. (2023). Only Plant-Based Food Additives: An Overview on Application, Safety, and Key Challenges in the Food Industry. Food Rev. Int..

[124] Chmelík Z., Kotolová H., Piekutowská Z., Horská K., Bartosová L., Suchý P., Kollár P. (2013). A Comparison of the Impact of Amaranth Flour and Squalene on Plasma Cholesterol in Mice with Diet-Induced Dyslipidemia. Berl. Munch. Tierarztl. Wochenschr..

